# Development and Testing of the Novel Sugar Meter for Informing Sugar Intake Guidelines to Parents of Three- to Six-Year-Old Children: A Cross-Sectional Study

**DOI:** 10.7759/cureus.47409

**Published:** 2023-10-20

**Authors:** Jeyshree Kannan, Ashwin M Jawdekar

**Affiliations:** 1 Paediatric and Preventive Dentistry, Bharati Vidyapeeth Dental College and Hospital, Navi Mumbai, IND

**Keywords:** sugar consumption, sugar frequency, who sugar guideline, dietary sugars, dental caries

## Abstract

Introduction: In recent years, the consumption of refined sugar has increased manifold. Added sugar is implicated in dental caries, cardiovascular risk and obesity amongst other conditions. The 1025 World Health Organization (WHO) Sugar Guidelines recommends sugar intake below 10% of energy, but there is limited awareness about the same in the general population. The aim of this study was to test the Novel Sugar Meter for informing the WHO Sugar Guidelines to the parents of three- to six-year-old children.

Methods: Twenty consenting parents and their three- to six-year-old children from an English-medium school in Navi Mumbai, India, were selected. The parents were asked to record their child’s baseline dietary data for three consecutive days including one weekend day. The Novel Sugar Meter, an indigenously developed ready reckoner for identifying the quantity of sugars consumed, was used. The parents were counselled using the Novel Sugar Meter (intervention) and standard instructions on the WHO guidelines (control). The dietary data were recorded again to assess and compare dietary behaviour modifications.

Results: Comparison of pre-intervention versus post-intervention sugar consumptions showed a statistically significant reduction in the Novel Sugar Meter group (t(10) = 3.70891; p = .001388) but not in the control group (t(10) = 0.94081, p = 0.35926). Both groups showed a reduction in the frequency of daily sugar exposure, with significantly more reduction in the Novel Sugar Meter group (p = .000049).

Conclusion: Novel Sugar Meter-based counselling has the potential for application for reducing the quantity and frequency of sugar consumption in children.

## Introduction

Sugar has been a part of our diet since time immemorial. Our ancestors could only consume sugar from fruits during harvest, which would typically span over a few months each year. In the Indian context, sugar has enjoyed popularity in the form of jaggery too. Added sugars (commonly referred to as free sugars) are known to be a leading cause of dental caries [1]. They have been implicated in increased cardiovascular risk, obesity/overweight, reduced dietary diversity and poor nutrient intake [2].

Sugary foods and beverages are known to be a leading cause of non-communicable diseases, such as obesity and dental caries in early childhood, which is considered a serious public health problem in both developing and industrialised countries. The overall prevalence of early childhood caries in India has been found to be 49.6% [3]. The WHO 2015 Sugar Guidelines strongly recommend reducing free sugar intake to <10% of the total energy intake with a conditional recommendation of reducing free sugar intake to <5% of the total energy intake [1]. However, awareness of the same in the Indian suburban population is questionable.

An increasing trend in the consumption of marketed foods high in free sugar content by children has been noted [2], which may be attributed to either a lack of awareness about the recommended limit of sugar intake or a lack of a tool to assess the daily free sugar intake. Although there are many databases available to assess the macronutrient composition of commonly marketed foods, there are none to assess the ‘free sugars’ present in these foods. A ready reckoner can be a handy tool for identifying and inferring daily free sugar intake from marketed foods. The aim of the study was to test the Novel Sugar Meter for informing the WHO Sugar Guidelines to the parents of three- to six-year-old children. The objective was to compare the consumption quantity and frequency of consumption of free sugars before and after the intervention.

## Materials and methods

Study setting and sample selection

This cross-sectional study was carried out by the Department of Paediatric and Preventive Dentistry, Bharati Vidyapeeth Dental College and Hospital, Navi Mumbai. The sample size was selected as 20 for this study, in accordance with a previous study [4]. After gaining requisite permissions from the head of the institute, three- to six-year-old students of an English-medium school in the suburban area of Navi Mumbai and their parents were recruited in this study. The inclusion criteria was healthy children aged three to six years, whose parents gave consent for participation in the study, whereas the exclusion criteria was children with medical conditions, acute dental conditions, dietary restrictions or food allergies and those who have recently undergone dental treatment.

The study was approved by the Institutional Ethics Committee of Bharati Vidyapeeth Dental College and Hospital (approval no. IEC262022021). Appropriate written permissions were sought from the principal of the school. The principal investigator informed the parents about the study, and voluntary participation was encouraged. The willing parents were provided with an information sheet detailing the outline of the study, and informed consent was obtained from the parents after clarification of any lingering doubts or misgivings.

Development of the Novel Sugar Meter

Sugar meter as a tool to track total sugar consumption was initially developed by Jawdekar and colleagues in 2016. The original sugar meter simplified the massive amount of information on food labels to user-friendly data. The authors concluded that the sugar meter could decipher complex labelling information and help people at large to make informed dietary decisions. Since the year of its development, changes to the market and regulations were anticipated and as such a need for updating the original sugar meter was felt. Food products containing free sugars that are most commonly marketed and consumed by the age group of three- to six-year-old children were identified and categorized under broad headings. The process of data collection included physical market surveying and surveying online retail platforms to maximize product diversification and inclusion. No additional external help was sought during the data collection. A total of 140 products were identified and classified under 12 categories. The Novel Sugar Meter was made available as a poster that can be printed and distributed.

Assessment of dietary behaviours

**Figure 1:**
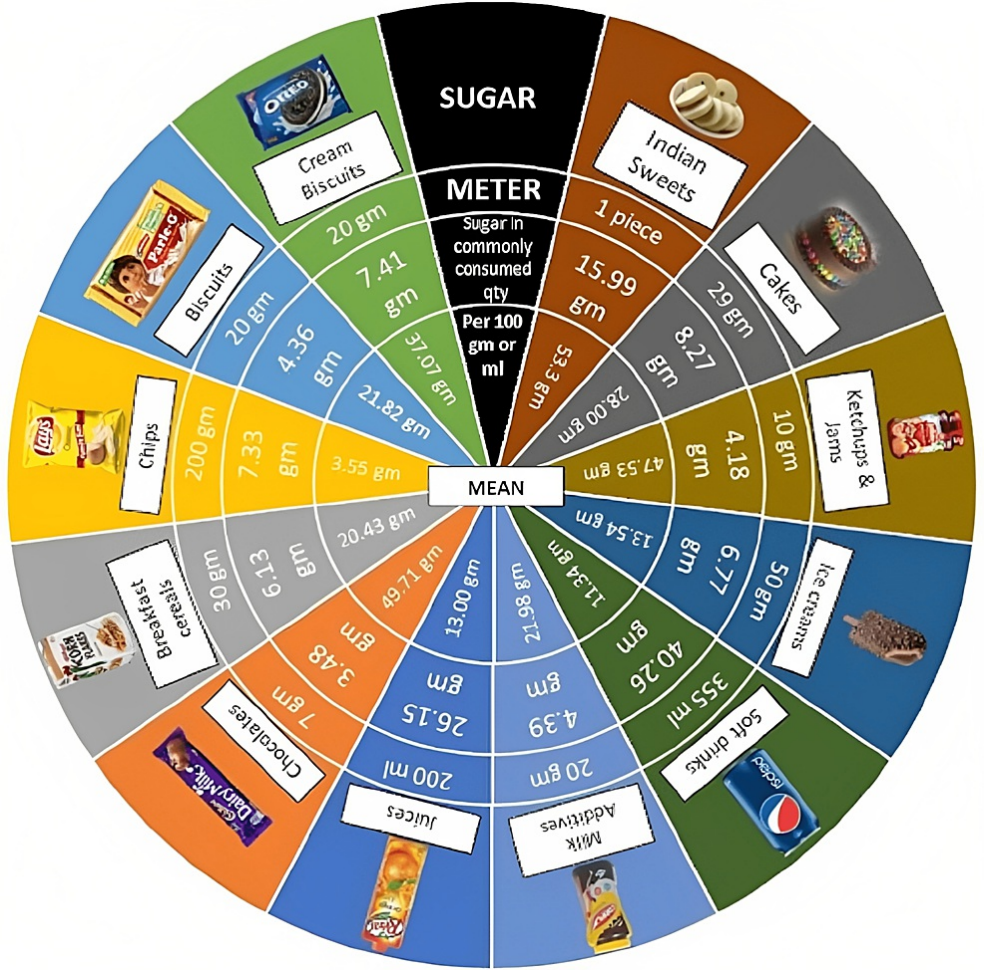
Novel Sugar Meter Image created by the authors

The parents were asked to record their child’s dietary data in a food diary for three consecutive days including one weekend day. Net sugar consumption was calculated. The parents were later divided into two groups: One group was informed of the recommended daily sugar intake with the help of the 2015 WHO Sugar Guidelines (control group). The other group was informed the same but with the help of the Novel Sugar Meter, as illustrated in Figure 1, wherein an interactive session on the use of the Novel Sugar Meter was conducted for the parents. Following the information/education of the parents, dietary data in a food diary for three consecutive days including one weekend day was collected yet again to assess any dietary behaviour modification. A detailed description of the differences between the groups can be found in Table 1. A comparison of dietary data was made between the two groups.

**Table 1:** Differences between the Novel Sugar Meter group and the WHO standard instruction group

Novel Sugar Meter group	WHO standard instructions group
Informed consent was obtained.	
Pre-intervention data for three consecutive days was recorded (including one weekend day).	
Total sugar consumption in grams was calculated.	
WHO (2015) Sugar Guidelines were informed using the developed Novel Sugar Meter.	WHO (2015) Sugar Guidelines were informed verbally.
The Novel Sugar Meter poster was provided to the parents to be used to record sugar consumption.	
Post-intervention data for three consecutive days were recorded (including one weekend day).	
Total sugar consumption in grams was calculated.	

Statistical analysis

The normality of data was verified using the Kolmogorov-Smirnov test, and the data were found to be normally distributed. Descriptive variables were recorded as the mean and standard deviation for the quantity and difference in the quantity of sugar consumed before and after intervention in both groups. The frequency of consumption was encoded as less than or equal to two exposures daily and more than or equal to three exposures daily. The frequency was calculated with the help of the mode. Tests, such as Student’s t-test and chi-squared test, were used for inferential statistics.

## Results

**Figure 2:**
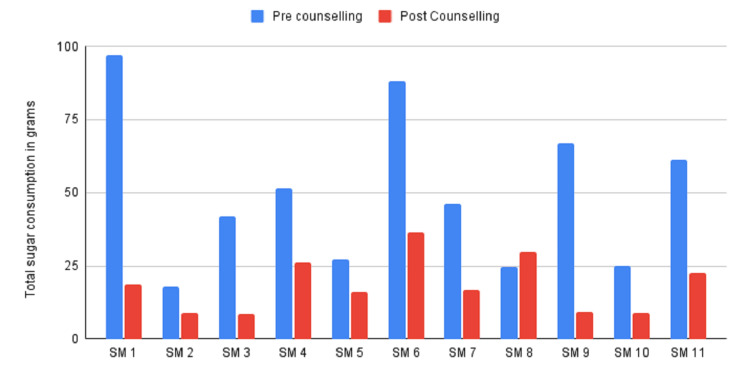
Pre-intervention versus post-intervention sugar consumption data for the Novel Sugar Meter group

**Figure 3:**
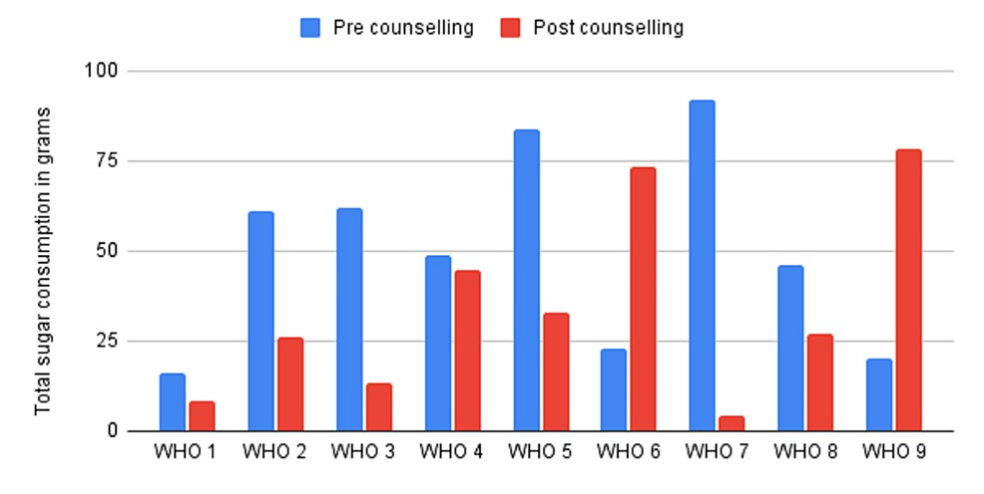
Pre-intervention versus post-intervention sugar consumption data for the WHO Sugar Guideline group

The two post-intervention groups were the Novel Sugar Meter group and the WHO group. The comparison between the reduction of sugar consumption amongst the groups is illustrated in Figures 2, 3.

The data for the comparison of the pre-intervention versus post-intervention quantity of sugar consumed for the Novel Sugar Meter and the WHO groups showed a statistically significant reduction in the sugar consumed in grams in the group of parents who were counselled using the Novel Sugar Meter (t(10) = 3.70891, p = .001388) as compared to the parents of the group who were counselled using the WHO Sugar Guideline only (t(8) = 1.2573, p = .226688).

The data for the comparison of differences in the before and after intervention sugar consumption amongst the Novel Sugar Meter group and WHO Sugar Guideline group showed no statistically significant reduction in the sugar consumed in grams between the groups (t(10) = 0.94081, p = 0.35926).

The frequency of sugar consumption at baseline did not differ for the two groups, with both showing a 70% frequency of ≤two sugar exposures per day. However, in the post-intervention assessment, 96.96% of children in the Novel Sugar Meter group showed ≤two sugar exposures per day as compared to 77.77% children of the WHO standard instruction group (p = .000049). This difference was statistically significant, as shown in Table 2.

**Table 2:** Comparison of the frequency of sugar consumption before and after intervention amongst the two groups * Novel Sugar Meter group; # WHO Sugar Guideline group; ^ significant for p < 0.05

Sugar exposure frequency	Pre		Post		Chi-square and P value
Categories	< /=2	>/= 3	= 2	>/= 3	16.502 (p = .000049^)
SM*	23 (69.69%)	10 (30.30%)	32 (96.96%)	1 (03.03%)	
WHO#	19 (70.30%)	8 (29.62%)	21 (77.77%)	6 (22.22%)	

## Discussion

As the Novel Sugar Meter aims for a quantitative reduction in the amount of sugar consumed, it reiterates the Mumbai Declaration on Sugary Drinks and Healthy Food released in June 2022 [5]. The Mumbai Declaration calls for seven key areas of action, amongst those is “creating an environment to adopt the WHO Guideline on Sugar.” The present study underlines that using a tool like the Novel Sugar Meter can significantly help reduce the quantitative burden of sugar intake per day.

The addition of sugars to packaged and processed foods has long been unregulated despite the initial attempts by government bodies [1,6-8]. Added sugars are known to play a pivotal role in caries incidence [2,9-13]. Unfortunately, evidence exists about the influence of the sugar industry on academic research [14]. It was found that the general public at large was still unaware of the intricate details of what was being proposed towards the reduction of sugar consumption. The Novel Sugar Meter was tested in a cross-sectional study format to validate its use in the current investigation.

The specific sample frame of an urban setting near a metropolitan city comprising parents of children in the three-to-six-year age group was chosen because children in this age group are easily accessible. By this age, all the primary teeth would have erupted, and more or less the dietary habits would have been established. Any intervention to reduce sugar consumption could have a protective effect on the permanent dentition to follow.

The time chosen for the study was that after Diwali, and care was taken to avoid festive occasions possibly leading to a significant rise in daily sugar consumption.

The aim of the study was to assess if the information presented in the form of the Novel Sugar Meter was more effective than knowledge dissipation using the 2015 WHO Sugar Guidelines alone. For this, the use of validated self-reporting methods (diet diary) was incorporated in this study. Ortega et al. (2015) reported the effective use of dietary records and recommended a minimum of three days to gain maximum information [15]. It has been reported by Gersovitz et al. (1978) that in the use of dietary records for more than four days, the recall is prone to the flat-slope syndrome [16]. The validity of dietary data can decline by the fifth, sixth, and seventh record days. Moreover, by the seventh day, the demographic nature of the sample may become biased due to drop-outs and decreased usability of the records. Regardless of the length of the dietary record, it is desirable to count in both working and weekend days, in order to get a better picture of the overall diet [15]. In keeping with the recommendations above, it was decided to use a three-day diet diary that included at least one weekend day to record the data with maximum accuracy.

The assessment of the validity of the Novel Sugar Meter was undertaken as a pilot study with a sample size of 20 as per the paper by Julious [4] who recommended a maximum sample size of 12 per group, beyond which there is no marked gain in the positive precision of mean and variance in parallel group studies, with a significant gain of positive precision sharply tapering off below 0.1 units for a sample size above 8. As such, for maximum precision, a sample size of above 8 was ideal, so a total of nine and 11 samples were taken for the Novel Sugar Meter group and WHO Sugar Guideline group, respectively. It is a preliminary study and a suitable small sample was considered.

The results show a significant reduction in the quantity of sugar consumed, as reported by the parents, in the Novel Sugar Meter group when compared to the pre-intervention levels. While the comparison between the groups for the difference in sugar consumption showed no statistically significant difference, this may well be attributed to the small sample size of the study.

The Novel Sugar Meter, in keeping with the 2015 WHO Sugar Guidelines, is intended to reduce the quantity of sugar intake. Meanwhile, the Sugar Clock proposed by Dhingar et al. in 2020 [17] addresses only the frequency of sugar consumption; in our study, the frequency of consumption was also found to be significantly lower post-intervention in the Novel Sugar Meter group as compared to the WHO standard instruction group.

The statistically non-significant difference for inter-group comparison for reduction in the quantity of sugar consumption could be either due to a small sample size or the control group performing as effectively as the Novel Sugar Meter. As for the sample size, this being a preliminary study, it was intentionally small. While the WHO standard instructions and Novel Sugar Meter were equally effective (which is the second inference of the non-significant difference in inter-group comparison) in reducing the quantity of sugar consumption, it is noteworthy that the Novel Sugar Meter is more effective in reducing the frequency of sugar consumption, which has been proven to be a major contributing factor in the etiopathogenesis of dental caries. Additional advantages of the Novel Sugar Meter are the pictorial nature coupled with its portability and potential in reinforcing behaviour changes that cannot be overlooked.

While the industry may drag its legs on the implementation of policies in line with the WHO Sugar Guidelines, the parents of children in the three-to-six-year age group may play a significant role in influencing their future sugar-related behaviours. It has been proven that trained non-parental persons are better at controlling sugar intake in children [18]. Therefore, more emphasis has to be laid on educating parents in order to effect sustainable change in sugar-related behaviours.

The frequency of sugar consumption was also calculated in this study. It was found that the frequency of consumption before intervention in the Novel Sugar Meter group reduced from more than or equal to three exposures to less than or equal to two exposures after the intervention. There was a rise in less than or equal to two episodes of sugar consumption per day after the Novel Sugar Meter was introduced.

The limitations of the study include the fact that the sample was suburban settings. Factors, such as the availability of products in the local market and purchasing preferences, may be different in different settings. The availability of certain products may differ from the suburban to the rural to a predominantly metropolitan population. The locale may also affect the preferences of a community, and as such, more such studies will help validate the Novel Sugar Meter for a wider population.

An accurate picture of sugar consumption was still not possible to record since the study does not account for all forms of sugar consumed other than those mentioned in the Novel Sugar Meter, such as in the form of jaggery, honey or added table sugar.

The non-probabilistic nature of the sample along with the possibility of the Hawthorne effect may have played a role in the overall results. The Hawthorne effect is where a person may change his or her behaviour just because they are being observed [19]. Despite its limited generalizability, there has been no such interventional study on counselling to reduce the sugar consumption of three- to six-year-old children. Dietary behaviour change is complex. Hence, more comprehensive research, addressing various issues, such as health literacy, availability of foods and cultural and societal factors, apart from counselling methods, is needed.

## Conclusions

The post-intervention reduction in the frequency of sugar consumption was higher in the Novel Sugar Meter group, and this was found to be statistically significant. In terms of the amount of sugar consumed, the Novel Sugar Meter group showed a statistically significant reduction, while the inter-group comparison was statistically nonsignificant. The Novel Sugar Meter may be handed over to parents or caregivers at public health promotion events and as a part of routine health education as a printout to be displayed prominently at home. Thus, the Novel Sugar Meter can serve as a useful tool for informing parents and facilitate behaviour change by effecting dissemination of the WHO Sugar Guidelines and provide guidance for sugar control.
